# Supplementary figures and images for: A chromosome-level sequence assembly reveals the structure of the Arabidopsis thaliana Nd-1 genome and its gene set
Source: PLoS One. 2019 May 21;14(5):e0216233. doi: 10.1371/journal.pone.0216233 (PMC6529160; doi:10.1371/journal.pone.0216233)

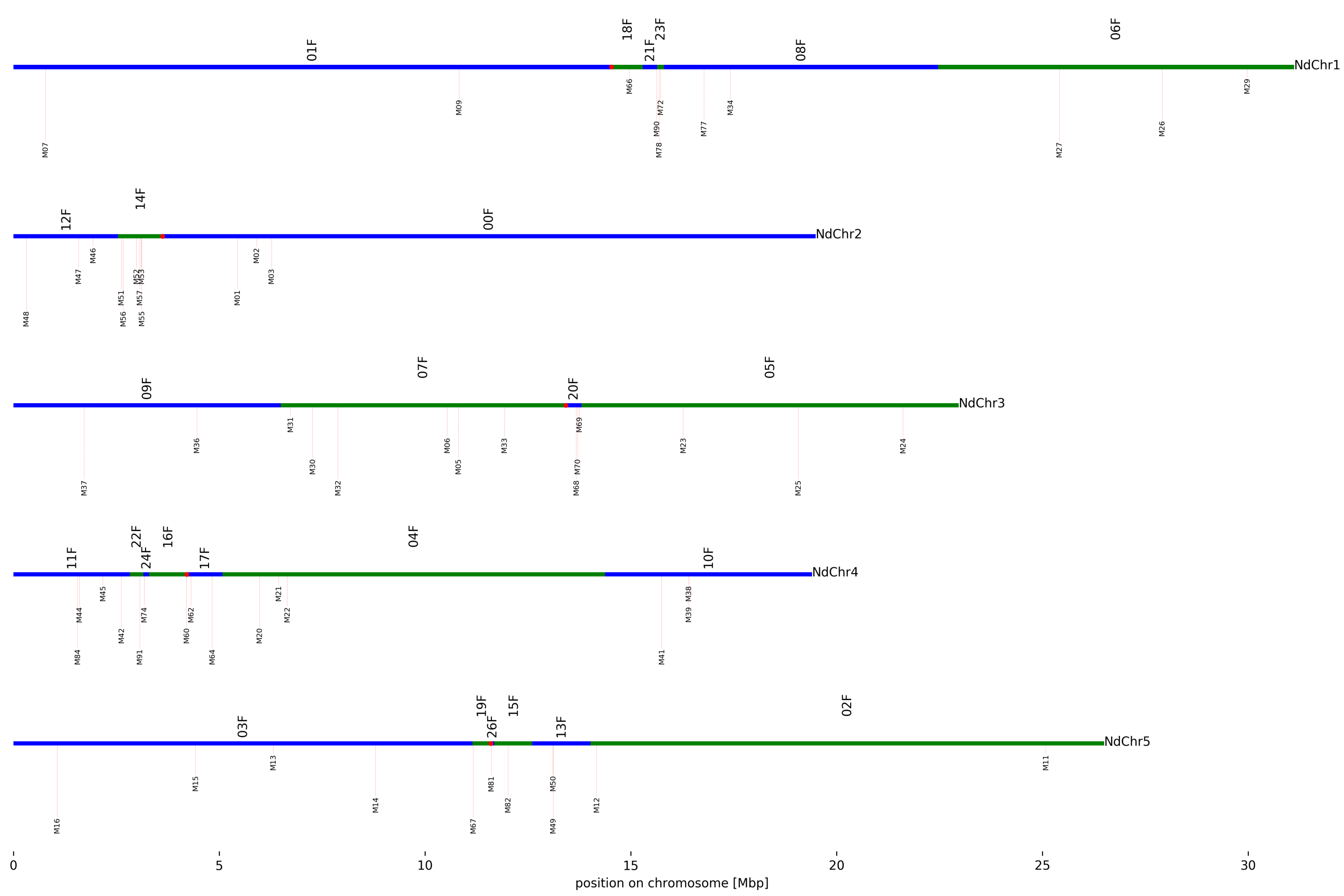

Supplement: S5 File — The positions of all genetic markers on the pseudochromosome sequences are illustrated. Assembled sequences were positioned based on the genetic linkage information. Some genetic marker combinations allowed the investigation of recombination frequencies within continuous sequences. (PDF) [file pone.0216233.s005.pdf]

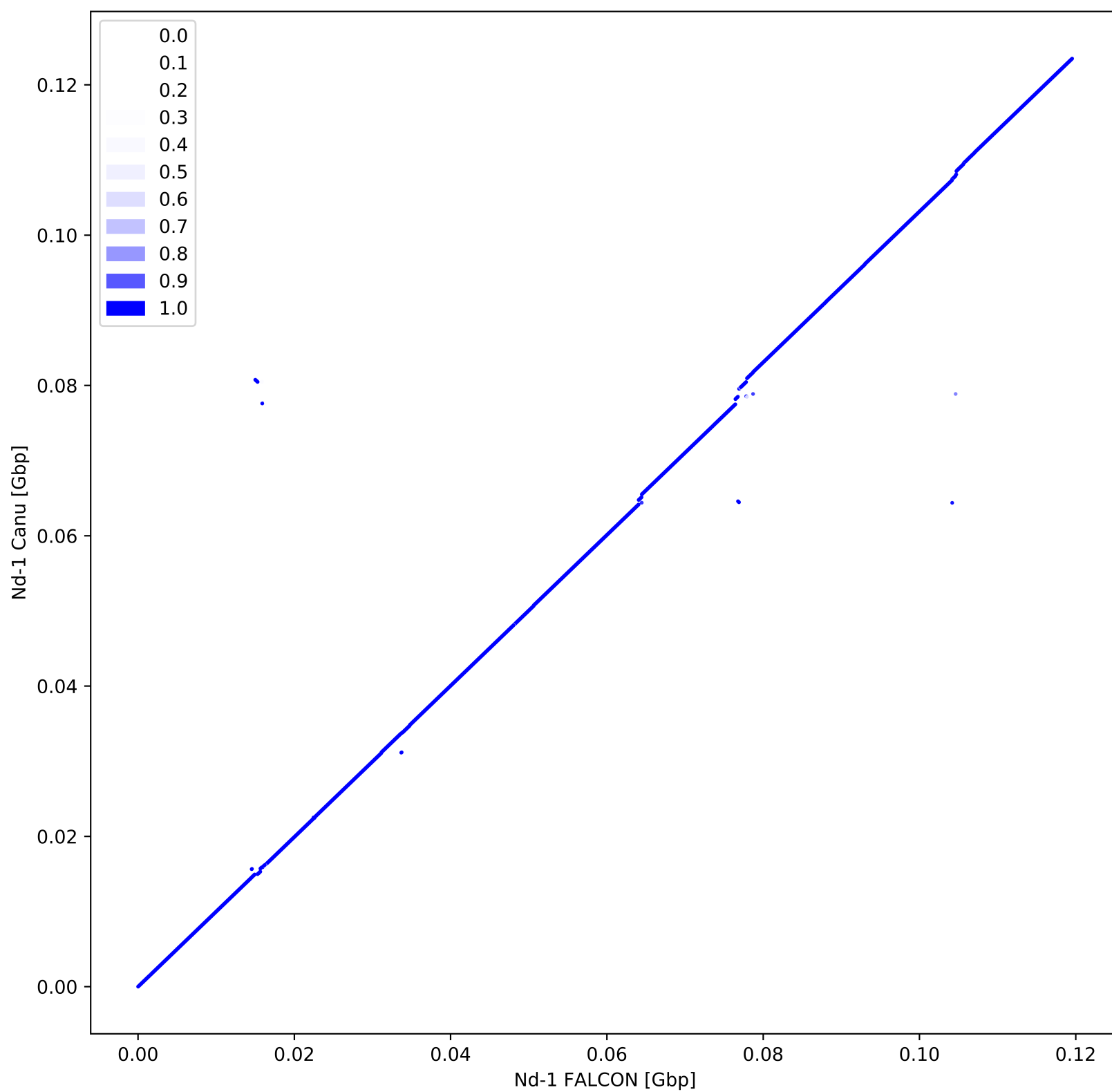

Supplement: S7 File — Assemblies generated by Canu and FALCON, respectively, were compared via BLASTn search of 10 kb sequence chunks. Color and position of dots in the figure indicate the position of the best hit on the respective sequence. (PDF) [file pone.0216233.s007.pdf]

Length: 154,443 bp

GC content  
GC skew+  
GC skew-

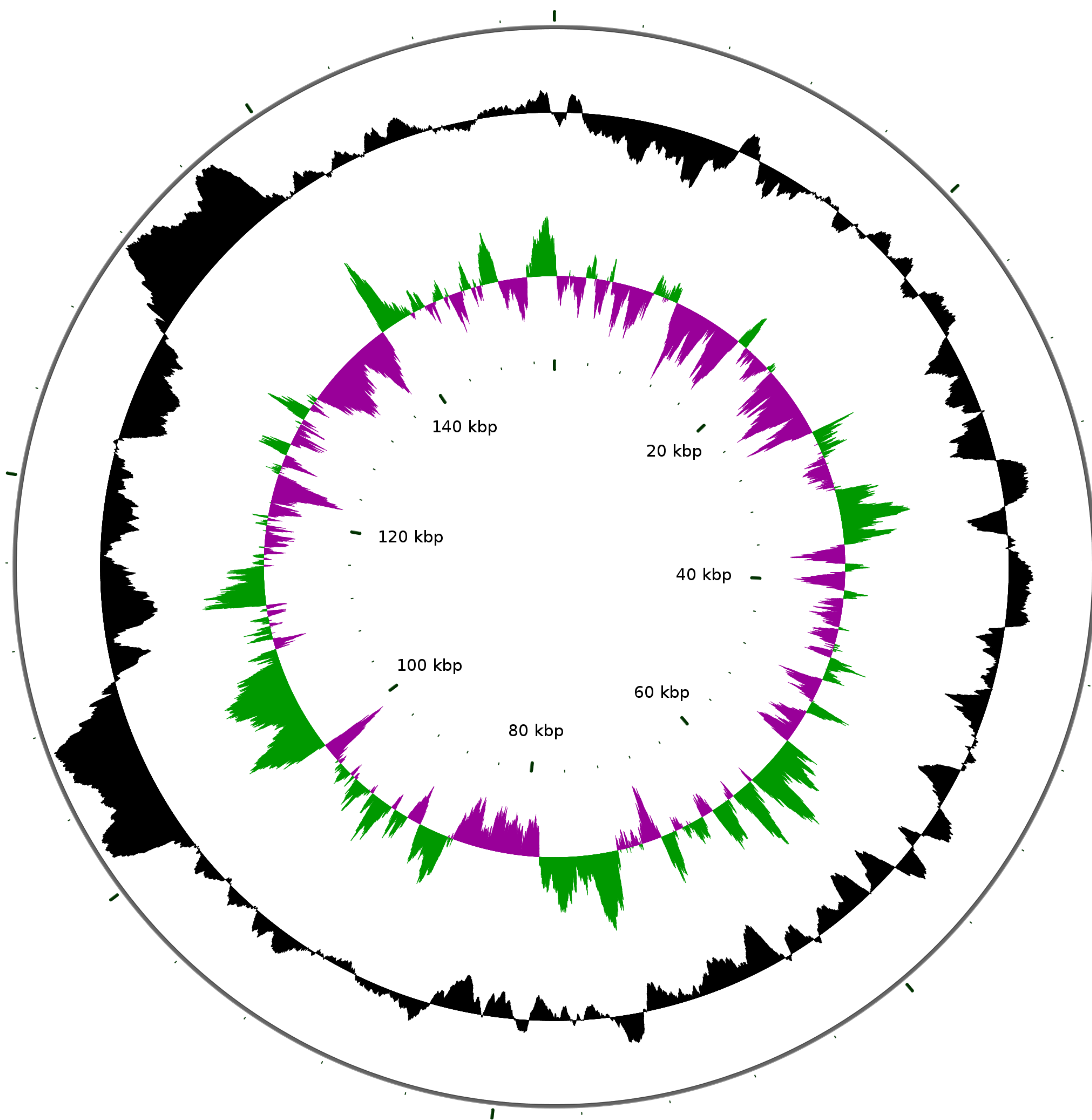

Supplement: S10 File — The GC content (black) and GC skew (green for positive GC skew, purple for negative GC skew) of the plastome sequence were analyzed by CGView [31]. The sequence and its properties are very similar to the Col-0 plastome sequence. (PDF) [file pone.0216233.s010.pdf]

Length: 368,216 bp

GC content  
GC skew+  
GC skew-

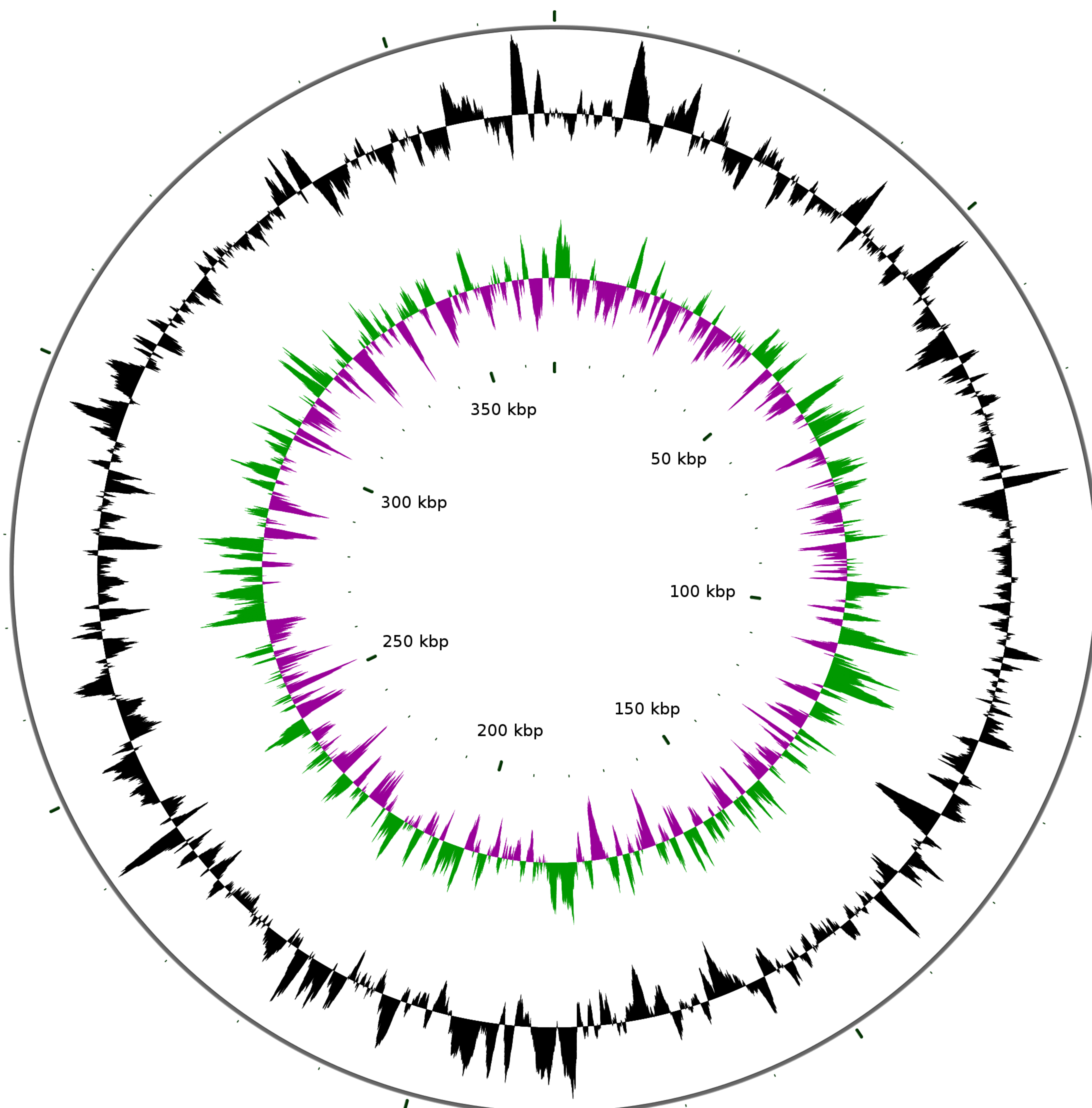

Supplement: S11 File — The GC content (black) and GC skew (green for positive GC skew, purple for negative GC skew) of the chondrome sequence were analyzed by CGView [31]. The sequence and its properties are very similar to the Col-0 chondrome sequence. (PDF) [file pone.0216233.s011.pdf]

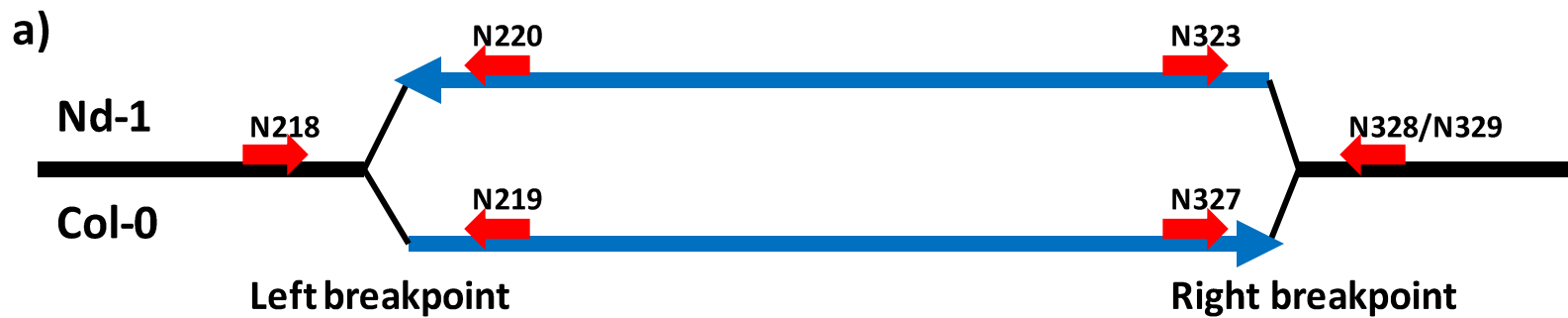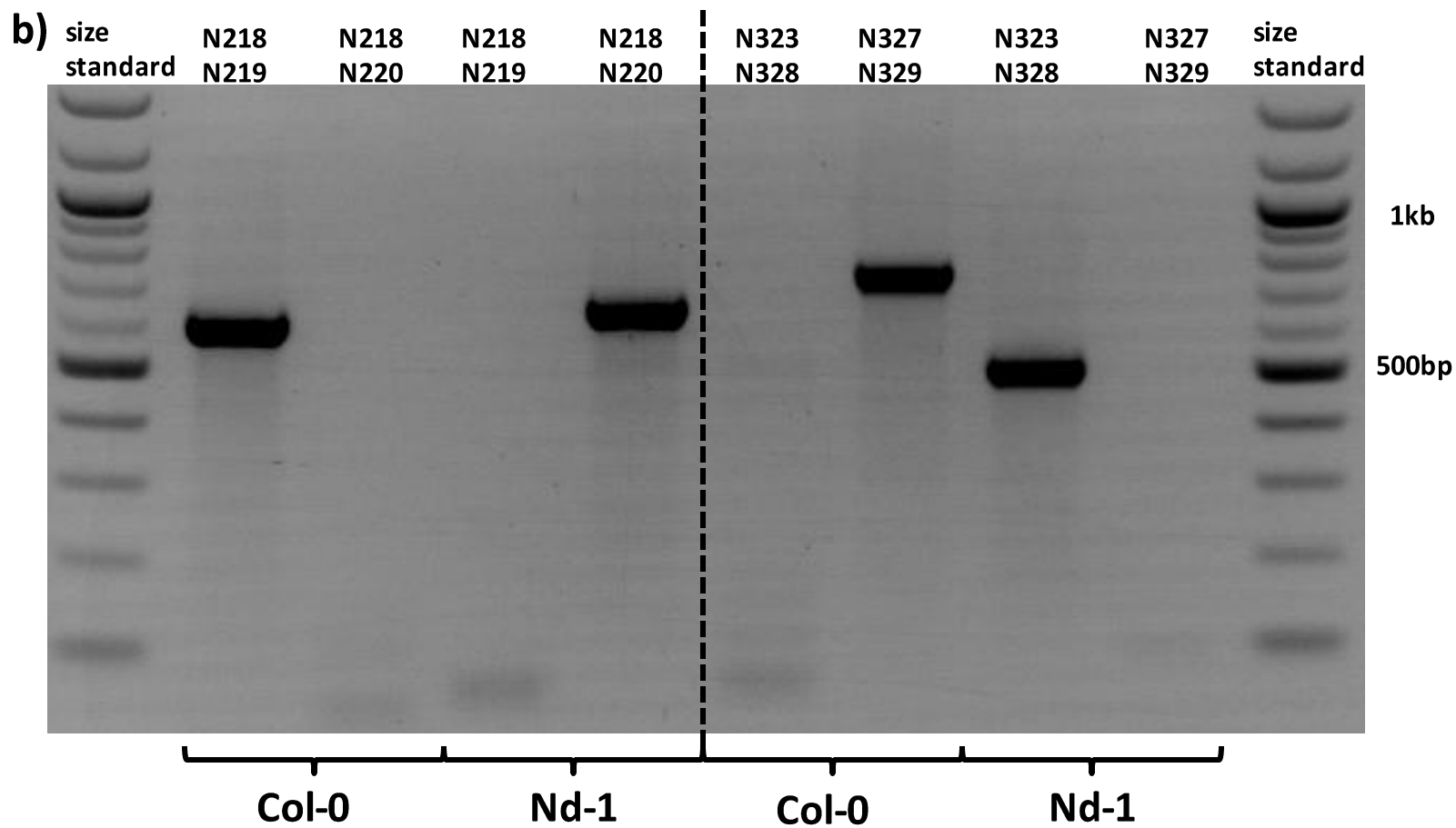

Supplement: S12 File — The identified inversion between Nd-1 and Col-0 on chromosome 4 is different from the inversion described before between Col-0 and Ler [14]. However, the left breakpoint is the same for both alleles enabling the use of previously published oligonucleotide sequences [14]. The right breakpoint was identified by manual investigation of sequence alignments. Both breakpoints were validated via PCR using the oligonucleotides (for sequences see S6 File) as illustrated in (a). The results support the expected inversion borders (b). (PDF) [file pone.0216233.s012.pdf]

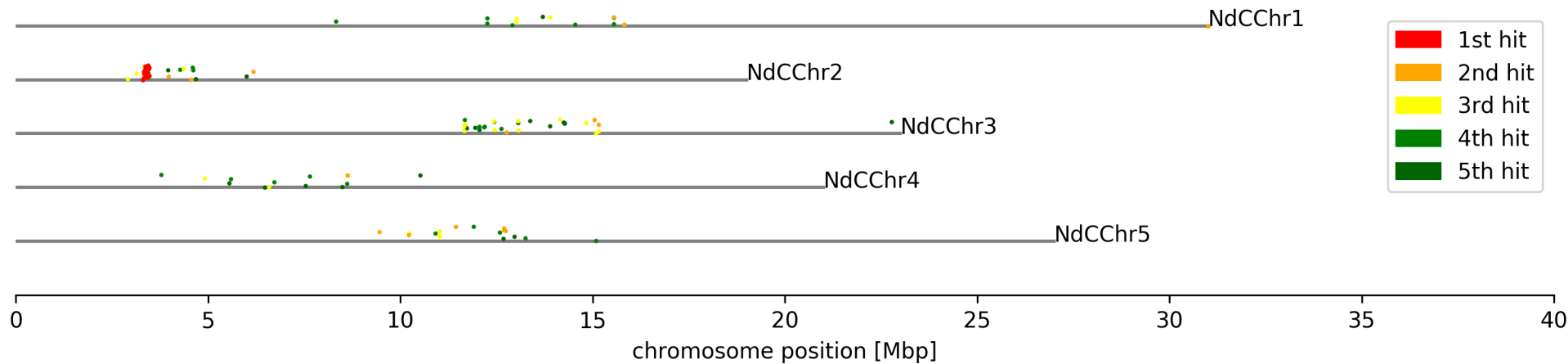

Supplement: S13 File — AthNd-1_v2c and the Col-0 gold standard sequence display a highly diverged region at the north of chromosome 2, which is about 300 kbp long. BLASTn of the complete Nd-1 gene sequences from this region revealed several regions on other Nd-1 chromosomes with copies of these genes. (PDF) [file pone.0216233.s013.pdf]

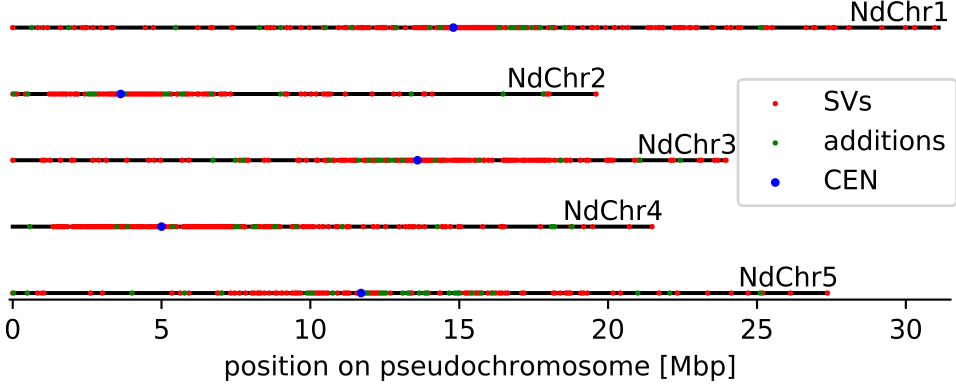

Supplement: S14 File — The distribution of structural variants (SVs) >10 kbp (red dots) between Col-0 and Nd-1 over all five pseudochromosome sequences (black lines) is illustrated. Additionally, the assumed centromere (CEN) positions are indicated (blue dots). Most SVs are clustered in the (peri-)centromeric region. (PDF) [file pone.0216233.s014.pdf]

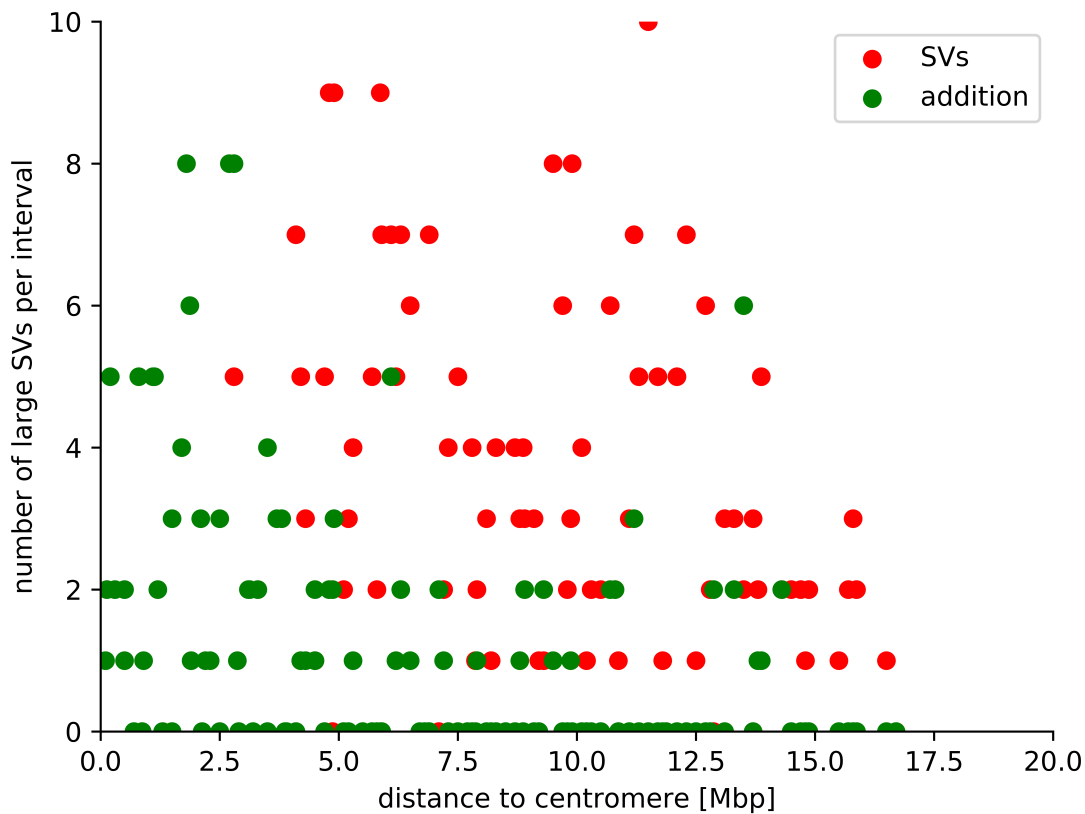

Supplement: S15 File — The correlation between the number of SVs in a given part of the genome sequence (1 Mbp) and the distance of this region to the centromere position is illustrated. SVs are clustered around the centromeres (Spearman correlation coefficient = -0.66, p-value = 1.7*10-16). (PDF) [file pone.0216233.s015.pdf]

Overlapping fraction of TEs and genes

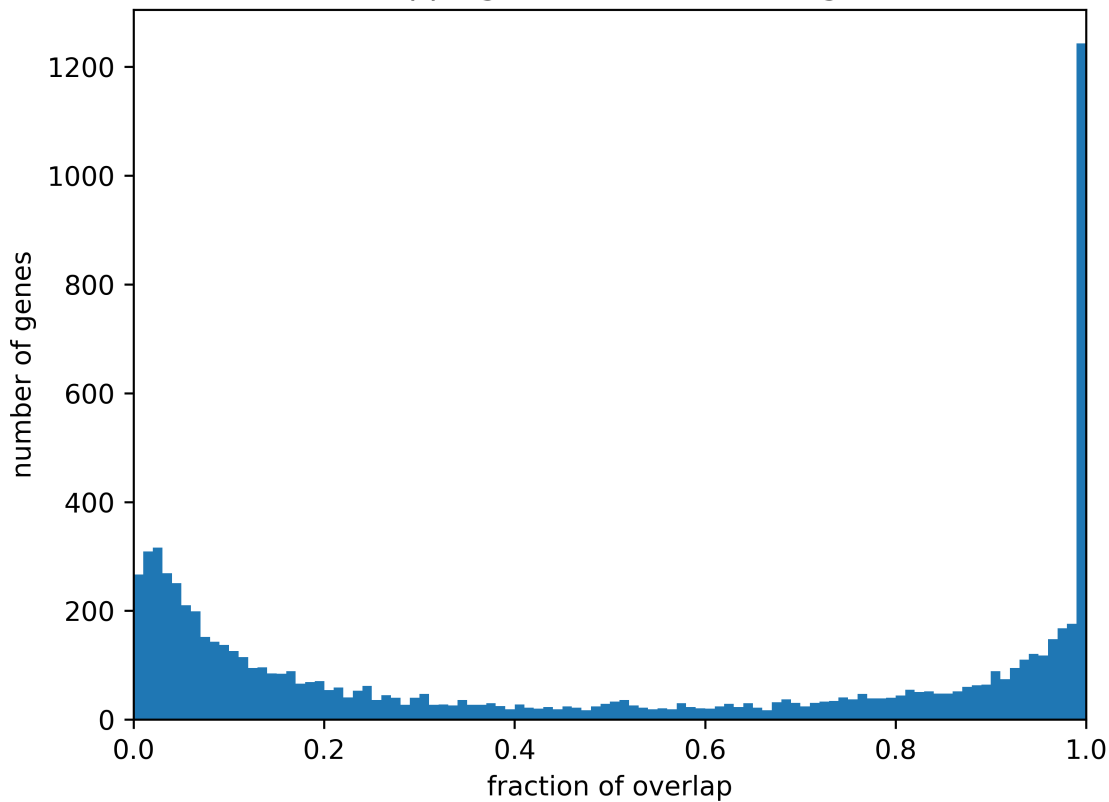

Supplement: S17 File — The overlap between annotated TEs (S8 File) and predicted protein coding genes was analyzed to identify TE genes. This figure illustrates the fraction of a gene that is covered by a TE. Since TEs might occur within the intron of a gene, only genes with at least 80% TE coverage were flagged as TE genes (S18 File). (PDF) [file pone.0216233.s017.pdf]

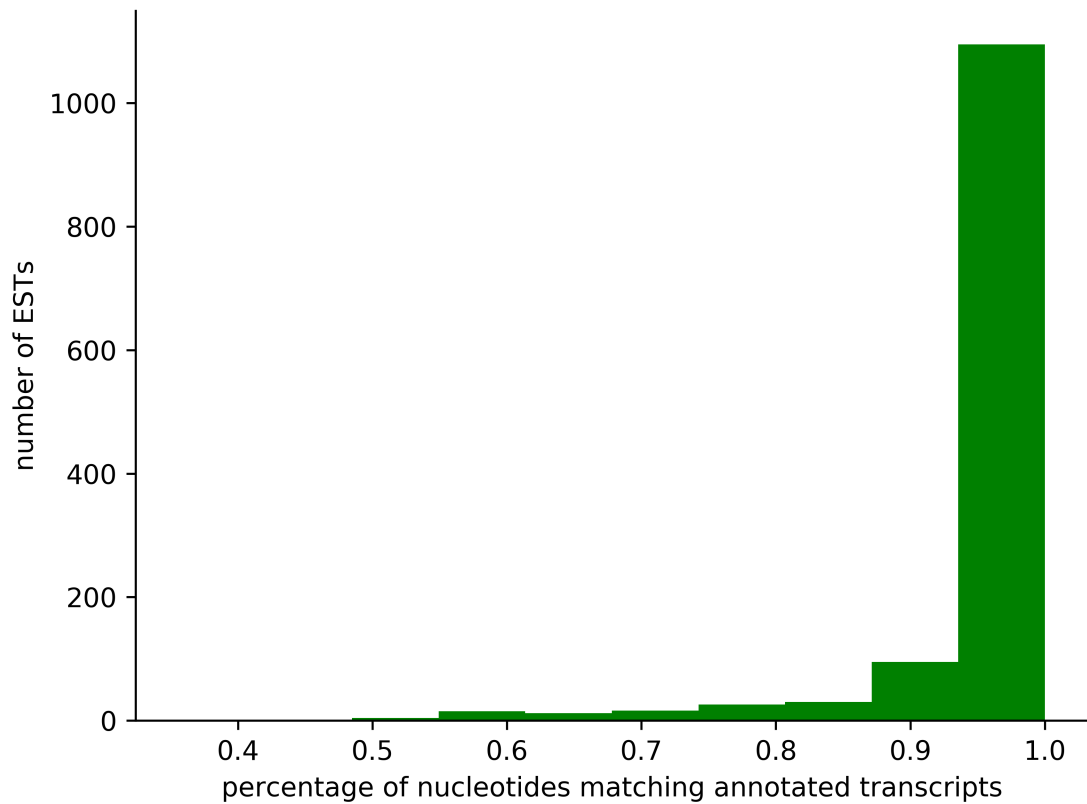

Supplement: S19 File — Percentage of nucleotides in ESTs matching predicted transcripts are displayed. (PDF) [file pone.0216233.s019.pdf]

Araport11

Nd-1\_v1.1

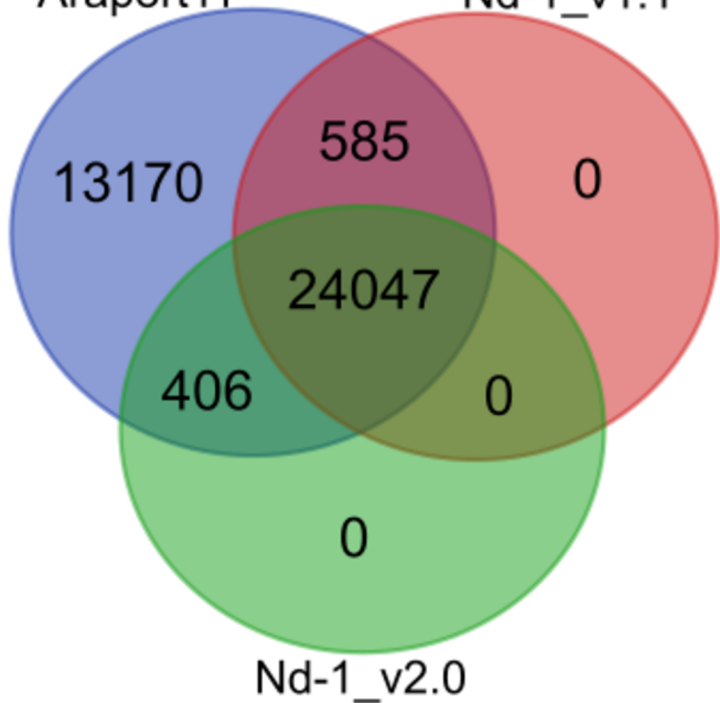

Supplement: S20 File — RBHs were identified pairwise between gene sets. The overlap was identified by mapping all genes onto Araport11 identifiers. Venn diagram construction was performed at http://bioinformatics.psb.ugent.be/webtools/Venn/. (PDF) [file pone.0216233.s020.pdf]

# RBH positions

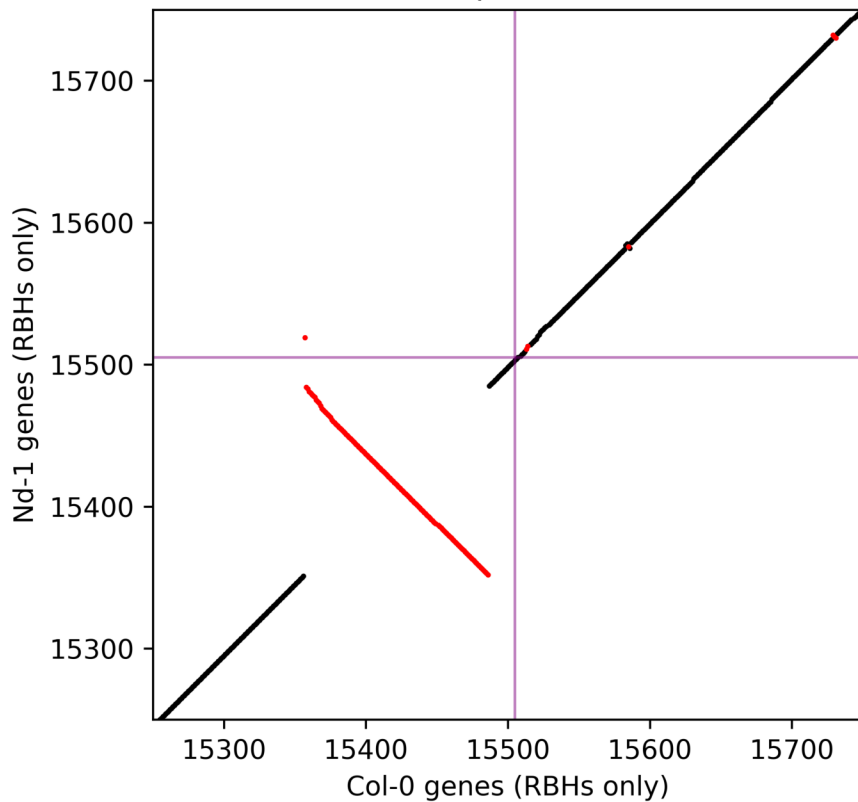

Supplement: S22 File — Genes in RBH pairs were sorted based on their position on the five pseudochromosomes of the two genome sequences to form the x (Col-0) and y (Nd-1) axes of this diagram. Plotting the positions of each RBH pair leads to a bisecting line of black dots representing genes at perfectly syntenic positions. Red and green dots indicate RBH gene pair positions deviating from the syntenic position. Red dots symbolize a unique match to another gene, while green dots indicate multiple very similar matches. Positions of the centromere (CEN4) on the chromosomes of both accessions are indicated by purple lines. An inversion involving 131 genes in RBH pairs just north of CEN4 distinguishes Nd-1 and Col-0. (PDF) [file pone.0216233.s022.pdf]

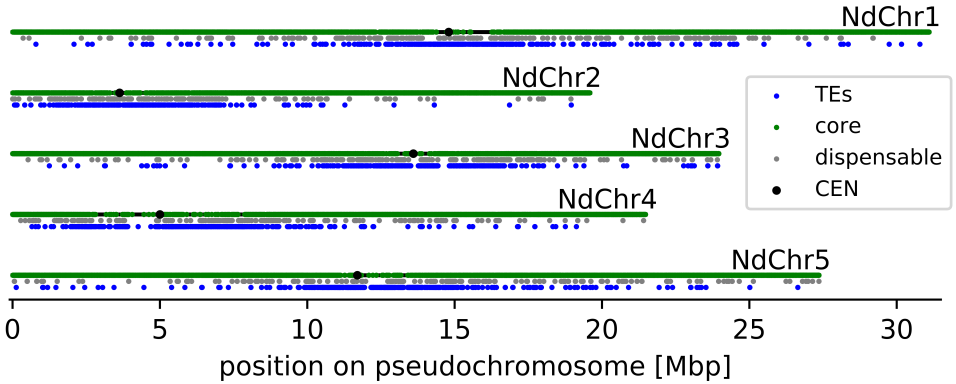

Supplement: S27 File — Visualisation of the position of core genes, dispensable genes and TEs along the chromosomes. (PDF) [file pone.0216233.s027.pdf]

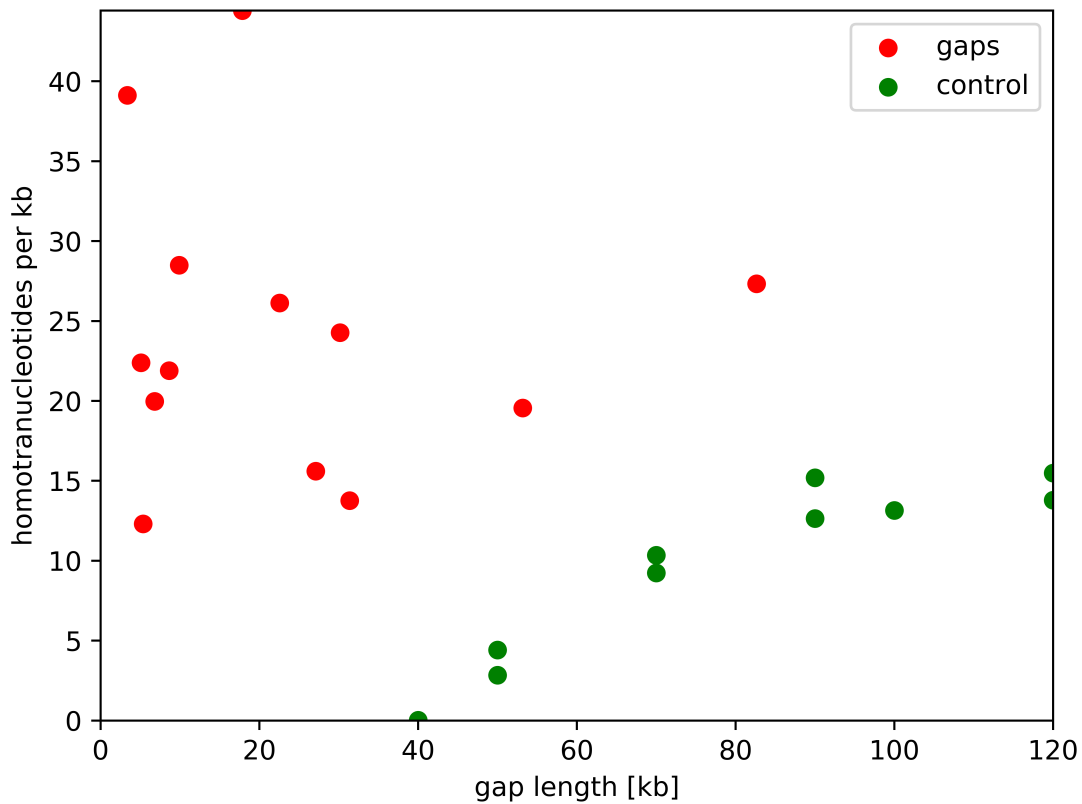

Supplement: S28 File — The high contiguity of the Ath-Nd-1_v2c assembly enabled the investigation of 13 sequences corresponding to gaps in the Col-0 gold standard sequence. This figure illustrates the homotetranucleotide occurrence in these sequences (red dots) in comparison to some randomly selected reference sequences (green dots). While there is a clear enrichment of homotetranucleotides in the gap-homolog sequences, there was no clear correlation between the length of a gap and the composition of the corresponding sequence observed. (PDF) [file pone.0216233.s028.pdf]
